# A Method to Assess Seasonality of Urinary Tract Infections Based on Medication Sales and Google Trends

**DOI:** 10.1371/journal.pone.0076020

**Published:** 2013-10-25

**Authors:** Louise Rossignol, Camille Pelat, Bruno Lambert, Antoine Flahault, Emmanuel Chartier-Kastler, Thomas Hanslik

**Affiliations:** 1 Département de médecine générale, UPMC Univ Paris 06, Paris, France; 2 UMRS 707, UPMC Univ Paris 06, Paris, France; 3 U707, INSERM, Paris, France; 4 U738, INSERM, Paris, France; 5 UMRS 738, Université Paris Diderot, Paris, France; 6 IMS-Health, La Défense, France; 7 Descartes School of Medicine, Sorbonne Paris Cité, Paris, France; 8 Urologist hopital universitaire Pitié-Salpêtrière AP-HP, faculté de médecine Pierre et Marie Curie Paris VI, Paris, France; 9 Université Versailles-Saint-Quentin-en-Yvelines, Versailles, France; California Department of Public Health, United States of America

## Abstract

**Background:**

Despite the fact that urinary tract infection (UTI) is a very frequent disease, little is known about its seasonality in the community.

**Methods and Findings:**

To estimate seasonality of UTI using multiple time series constructed with available proxies of UTI. Eight time series based on two databases were used: sales of urinary antibacterial medications reported by a panel of pharmacy stores in France between 2000 and 2012, and search trends on the Google search engine for UTI-related terms between 2004 and 2012 in France, Germany, Italy, the USA, China, Australia and Brazil. Differences between summers and winters were statistically assessed with the Mann-Whitney test. We evaluated seasonality by applying the Harmonics Product Spectrum on Fast Fourier Transform. Seven time series out of eight displayed a significant increase in medication sales or web searches in the summer compared to the winter, ranging from 8% to 20%. The eight time series displayed a periodicity of one year. Annual increases were seen in the summer for UTI drug sales in France and Google searches in France, the USA, Germany, Italy, and China. Increases occurred in the austral summer for Google searches in Brazil and Australia.

**Conclusions:**

An annual seasonality of UTIs was evidenced in seven different countries, with peaks during the summer.

## Introduction

Urinary tract infection (UTI) is considered to be the most common bacterial infection, with *Escherichia coli (E. coli)* involved in 70–80% of cases [1], [2]. It affects mainly women. By the age of 24, one third of women have experienced at least one UTI [3].

Seasonal variation in the incidence of *E. coli* bloodstream infection has recently been reported in different countries [4]. UTI is the primary source of these infections [5]. Despite this fact, little is known about the seasonality of UTIs in the community. If seasonality was evidenced, it could help to implement infection control interventions and to orient research along pathophysiological pathways [6], [7].

There is a lack of historical systems of UTI surveillance in the population. Indeed, to our knowledge, there is no available UTI incidence time series to test the seasonality of UTI in the community. Epidemiological studies based on microbiological databases are exposed to biases as many uncomplicated UTI are diagnosed and treated without microbiological confirmation. In such a context, non-clinical databases such as medication sales and web searches can be used for studying seasonal trends. They have already proved to be useful proxies for other diseases [8], [9], [10], [11], [12].

Here, we analysed multiple time series of medication sales and Google search data 1) to test if a seasonality of UTI exists or not, and 2) if seasonality exists, to determine its magnitude.

## Materials and Methods

### Ethical considerations

This study was conducted by the Sentinel network. The Sentinel network is regulated by the mixed research unit UMR-S 707 of INSERM and University of Paris VI: Pierre and Marie Curie, in collaboration with the French Institute for Public Health Surveillance (Institut de veille sanitaire, InVS). It has obtained a research authorisation from the French independent administrative authority protecting privacy and personal data (CNIL), n°471 393. This study does not involve human participants and specific ethical approval is not required for this study.

### Data

A database containing aggregated medication sales was provided by IMS-Health France, a private company collecting data from 59% of French community pharmacies [13]. Their medication sales data are provided for free, within an agreement between Inserm and IMS Health, and can only be used for scientific research. The provided data consisted of weekly aggregated sales of 593 therapeutic classes, following the Anatomical Classification of the European Pharmaceutical Marketing Research Association [14]. There are many therapeutic classes including antibiotics, many of them having several indications (i.e. fluoroquinolone can be used for UTIs or respiratory infections). However, therapeutic class G04A1 includes only antibiotics with no indication other than uncomplicated UTI in France [15]. The oral form of fosfomycin represented 97% of the sales in this class. This antibiotic is recommended by French guidelines as the first line treatment for uncomplicated UTIs in women [16]. The remaining 3% of the therapeutic class G04A1 was sulfamethizole (as single component substance), an antibiotic still prescribed only for patients with uncomplicated UTI [15], [17]. Thus, therapeutic class G04A1 represented a specific indicator of UTI. The IMS-Health database covered the period between 4 September 2000 and 2 September 2012. Sales were measured in numbers of boxes, per week, per 100,000 inhabitants.

Temporal trends in web searches containing either *cystitis* or *urinary tract infection* (or both) were downloaded from Google Trends [18]. This is a public web facility of Google Inc., showing how often a particular search term is searched for, relative to the total number of searches, in various countries and languages. The queries from eight countries were studied: Germany, France, Italy, the USA, Australia, South Africa, China, and Brazil (countries were picked in both hemispheres, with at least one on each continent and, for Europe, one in the north, one in the middle and one in the south). Queries were translated into the native language of the country: *cystite* and *infection urinaire* for France, *cystitis* and *urinary tract infection* for the USA, Australia and South Africa, *blasenentzündung* and *harnwegsinfektion* for Germany, *cistite* and *infezioni delle vie urinarie* for Italy, 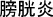 and 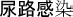 for China, and *cistite* and *infecção urinária* for Brazil. Each point of the time series represents the search fraction of the requested query over all queries made the same week in the country. Google Trends does not directly report this fraction but instead rescales it between 0 and 100, 100 being the highest point of the time series. The Google database covered the period between 11 January 2004 and 30 December 2012.

### Analysis

First, we calculated the mean and standard deviation (sd) of observations in the summer (*i.e.* week 25 to week 38, June to September) and the winter (i.e. week 51 to week 11, December to March), for each time series. Statistical differences were determined via the Mann-Whitney test. Then, seasonality was evaluated using the Harmonic Product Spectrum (HPS).

Most of the studied time series displayed some secular trends, often including a long-term tendency to increase. Thus, before looking for seasonal patterns, we estimated and removed these secular trends, using cubic smoothing splines [19]. The smoothing parameter was determined through generalised cross-validation, using the *mgcv* package of the R software package [20]. The frequency spectrum was obtained with Fast Fourier Transform (FFT) on medication sales data from 4 September 2000 to 2 September 2012, and on Google data from 11 January 2004 and 30 December 2012 [21]. We identified the fundamental frequency by applying the HPS method to the frequency spectrum of the de-trended time series [22].

## Results

All the time series could be analysed with the exception of South Africa, where the volume of search data was not reported before 2011. We thus discarded this series.

Annual peaks were in the summer for UTI drug sales in France and Google searches in France, Germany, Italy, the USA, and China. They were in the winter for Google searches in Brazil and Australia, *i.e.* during the austral summer for this southern hemisphere country. The mean (sd) of drug sales in France during the summer was 74.4 (13.0) boxes per 100,000 inhabitants per week. It was 62.2 (10.9) boxes per 100,000 inhabitants per week during the winter, that is, a 20% increase (p<0.001). UTI-related searches on Google were more frequent in the summer compared with the winter, with increases ranging from 8% to 19% (Table 1).

**Table 1:** Seasonal variations in the eight time series between summer and winter.

	Mean in winter (sd)	Mean in summer (sd)	Proportion of increase	p value, Mann-Whitney's test
Fosfomycin and sulfamethizole sales in France# (2000–2012)	62.2 (10.9)	74.4 (13.0)	20%	<0.001
Google searches* (2004–2011)				
France	53.7 (13.4)	61.7 (14.1)	15%	<0.001
USA	77.0 (7.0)	84.6 (6.4)	10%	<0.001
Germany	50.3 (12.7)	54.2 (10.9)	8%	<0.001
Italy	59.5 (10.6)	70.7 (12.9)	19%	<0.001
China	54.2 (11.7)	62.7 (12.1)	16%	<0.001
Brazil	30.8 (10.7)	27.1 (5.6)	13%	0.003
Australia	55.5 (16.3)	53.2 (9.0)	4%	0.01

# expressed as number of boxes per week per 100,000 inhabitants.

* Google searches containing the terms cystitis and/or urinary tract infection. Each is rescaled between 0 and 100 by Google Trends internal processes at download.

sd = standard deviation.

**Figure 1:**
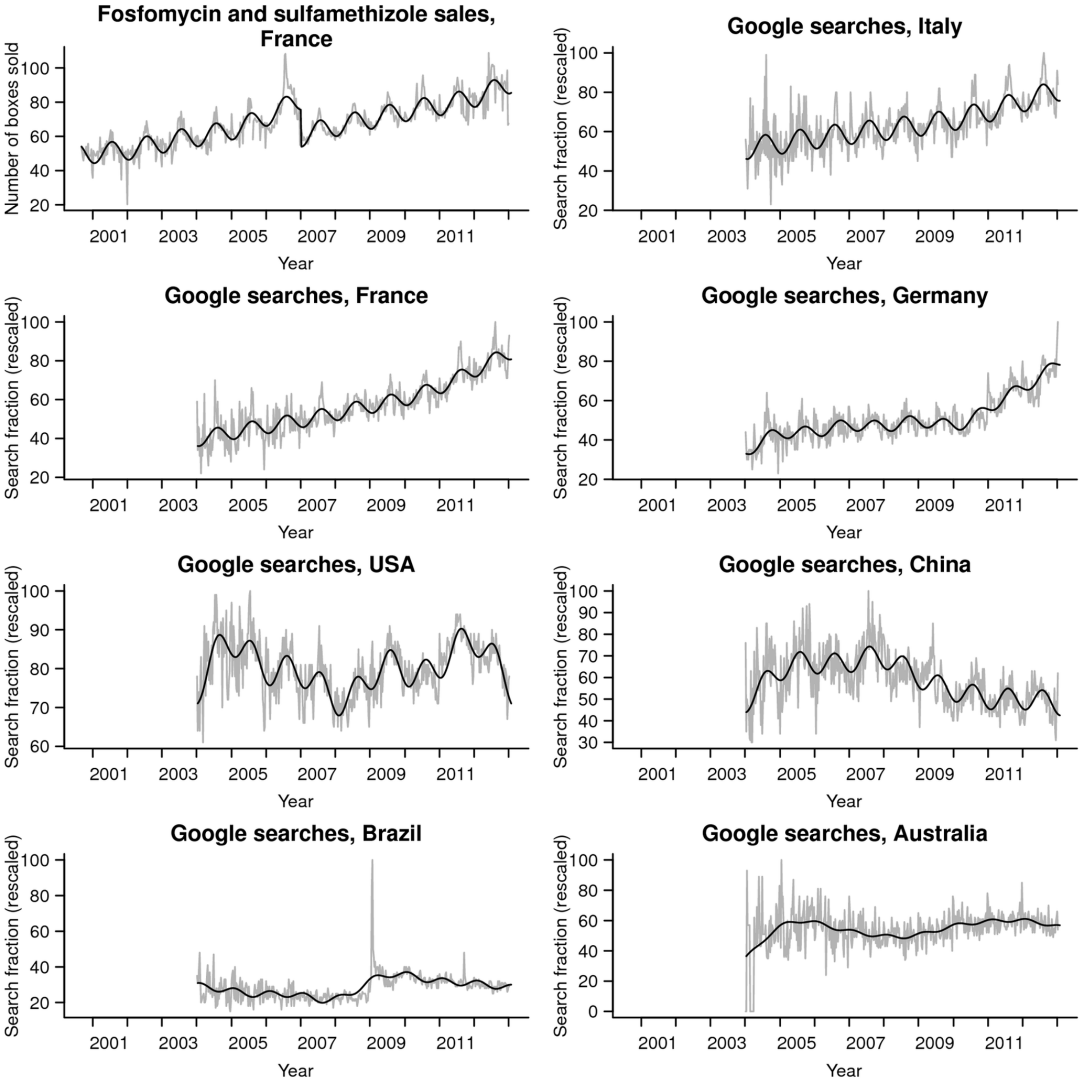
Time series of antibiotic sales and Google search queries for urinary tract infections. Using “fosfomycin and sulfamethazole” for antibiotic sales, 2000–2012, in France and “cystitis or urinary tract infection” for Google search queries, 2004–2012 in France, the USA, Brazil, Italy, Germany, China and Australia indicated in grey. Black lines show the model's predictions. The horizontal axis represents the time of year, the vertical axis for antibiotic sales represents drug sales (in number of boxes per 100,000 inhabitants), and the vertical axis for Google search queries represents search fraction. Search fractions are rescaled between 0 and 100 by Google Trends internal processes at download.

The fundamental period found by HPS was one year for the eight series: 52.17 weeks for sales of the therapeutic class G04A1 in France, and 52.22 weeks for the Google search series in France, Italy, Germany, the USA, China, Brazil, and Australia. Figure 1 plots the one-year pattern fitted to all time series: it clearly captures the major seasonal variations.

## Discussion

This study provides evidence of the seasonality of UTI, based on specific non-clinical indicators. Peaks were in the summer in France, Germany, Italy, the USA, and China, and during the austral summer in Brazil and Australia. The summer increase ranged from 8% to 20%, compared to winter periods. The fundamental frequency analysis conducted here was not hypothesis-driven, yet consistently returned a 52-week fundamental period for all series.

The seasonality of UTIs was first suspected in Canada [23]. The authors conducted their study in a specific population, and only culture-positive cases of UTI were included. Also, the method used did permit an estimate of the magnitude of the seasonal variations. Time series analyses were later performed for bloodstream infections in different countries [5], [24], [25], [26]. All these studies suggested seasonality of *E. coli* bloodstream infections, with summer increases ranging from 12% to 35% compared to the winter or to the rest of the year. No explanation was provided to explain the summer increases. In some previous studies, it was shown that higher temperature was associated with increased frequency of *E. coli* bloodstream infections [5], [24], [26]. Other hypotheses than the effect of temperature have been proposed, such as reduced micturition due to relative dehydration or more frequent sexual activity due to behavioural changes in the summer [27]. Divergent results have been found, for community-acquired infections caused by extraintestinal pathogenic *E. coli*, including UTI in children, with peak periods during the winter months [28], [29]. Such discrepancy could be explained by the fact that these studies did not include UTI only, but also any other kind of *E. coli* infections, or within specific populations (i.e. children). This could result in different pathophysiology and thus in different epidemiology.

During 2011, Google modified their geographical assignment, with retroactive application from 01/01/2011 [30]. Search trend data being given as fraction of the total search volume, this change in geographical assignment would have an impact only if the new defined areas were very discrepant from the old ones in terms of search patterns. This did not appear to be the case, as no irregularity like a sudden increase or drop in mean search fraction was noticed around the date of 01/01/2011 on the time series we downloaded. Also, during 2011 Google updated its categorization taxonomy [31]. However, this did not affect our data since we did not use categories when specifying our queries of interest, but used only keywords.

It is noteworthy here that consistent results were obtained from two different data sources: medication sales and Google search trends, and from seven different countries. When no historical systems of disease surveillance in the population are available, such proxies have already been validated for studying seasonal trends of other diseases, such as gastroenteritis, influenza, dengue, and depression [8], [9], [10], [11].

This study has several limitations. First, we supposed that there was a stationary link between the incidences and the proxies we used. However, regarding the Google searches, any publicised event relating to the studied disease can prompt searches, as has been seen previously, for example, in the case influenza during the increase in avian flu cases in 2005 or during the 2009 pandemic [8], [32], [33]. In our study, the Google UTI searches in Brazil peaked in January 2009, in conjunction with the death of a Brazilian top model from a UTI, which was highly covered by the press [34]. The interpretation of Google searches must therefore always be cautious, and based on several years of observation to avoid such biases, as was done in the present study. Second, there may be a bias in the number of Google searches related, say, to the more frequent use of search engines in the summer. However, Google uses a method to rescale the fraction of searches. If people use search engines more in the summer, this is taken into account in their model. So, the time series reflected well the seasonal variation in specific queries. Moreover, we verified that the Google searches fit well with antibiotic sales in France, which provided reassurance on the pertinence of this proxy for studying seasonality (the lag was at two weeks, data not shown). Third, drug sales estimates were based on a therapeutic class that did not include all antibiotics given for UTI treatment. However, our aim was not to estimate UTI incidence, but only to test seasonality. Thus, completeness was not necessary here, as it was more important to have a specific indicator of UTI, as was the case with the therapeutic class used in this study. Fourth, we observed temporal trends in multiple UTI time series, which increased regularly between 2000 and 2012, either in drug sales or in Google searches. We have no explanations for these trends (the method we used does not permit us to conclude that they reflected an increase in the incidence of UTI). However, our analyses were adjusted based on these trends, which consequently did not alter seasonality estimation.

## Conclusions

To conclude, it was shown here that seasonality of UTI searches exists in various countries, resulting in increases in the summer. The magnitude of these increases has been estimated for the first time, to our knowledge. As UTI is a very frequent disease, any intervention that can help to decrease its incidence will have a useful impact. For example, a next step following this work would be to test control measures such as the reinforcement of UTI prevention measures during the summer.
